# Evaluation of the measurement properties of the *parental perceptions of children’s exposure to tobacco smoke* instrument translated and adapted to the Brazilian context

**DOI:** 10.1097/MD.0000000000040147

**Published:** 2024-10-25

**Authors:** Maria Alice Santos Tavares, Vicki Myers, Leandro Alberto Calazans Nogueira, Agnaldo José Lopes

**Affiliations:** a Post-Graduation Programme in Rehabilitation Sciences, Centro Universitário Augusto Motta (UNISUAM), Rio de Janeiro, Brazil; b Instituto Nacional do Câncer José Gomes de Alencar, Rio de Janeiro, Brazil; c Department of Health Promotion, School of Public Health, Faculty of Medicine, Tel Aviv University, Tel Aviv, Israel; d Gertner Institute of Epidemiology & Health Policy Research, Sheba Medical Center, Ramat Gan, Israel; e Physiotherapy Department, Instituto Federal do Rio de Janeiro (IFRJ), Rio de Janeiro, Brazil.

**Keywords:** assessment tools, community, factor analysis, family health, infant health

## Abstract

Given the need to measure parents’ perceptions of their children’s exposure to tobacco smoke, the *Parental Perceptions of Children’s Exposure to Tobacco Smoke* (PPE) instrument was developed and validated in Hebrew and later translated into English and Portuguese. The PPE has already been translated and adapted to the Brazilian context but a more robust sample is necessary to investigate the measurement properties. Thus, this study evaluated the measurement properties of the Brazilian version of the PPE instrument. Reliability and validity study were conducted with 161 parents with children up to 18 years of age. Perception of parental smoking status was investigated using analysis of variance. The instrument showed excellent reliability (Cronbach α = 0.92 and intraclass correlation coefficient = 0.91). The exploratory factor analysis identified 4 factors with a total accumulated variance of 71.6%. Confirmatory factor analysis validated this structure with 4 factors. It was necessary to exclude a question that presented low commonality. Analysis of variance demonstrated that nonsmoking and former smoking parents have similar scores, although they are higher than those of current smokers. Our findings provide evidence that the PPE version translated and adapted for the Brazilian context is a reliable and valid instrument. Thus, it can help us understand how parents cope with exposure to tobacco smoke.

## 1. Introduction

A large proportion of children are exposed to cigarette smoke, especially in their own home environment. Around half a billion children globally are thought to come into contact with secondhand smoke while at home.^[1]^ Although adults can choose to smoke, children who live, play, or study in areas frequented by smokers are exposed to the harms of passive smoking and third-hand smoke (THS).^[2,3]^

Passive smoking can be understood as a person who does not smoke but lives with a current smoker and, in a closed environment, is forced to breathe air containing the toxic and carcinogenic elements of tobacco.^[4,5]^ The severity of exposure depends on several factors, such as the size of the space where the exposure occurs, the number of people who smoke, the exposure time, the age of the passive smoker, the periodicity of air exchange, and the use of purifiers.^[6,7]^ Passive smoking is considered the third cause of preventable death in the world, behind only active smoking and alcoholism. The World Health Organization warns that around 50,000 children die annually from diseases associated with passive smoking.^[8]^

A new concept that has been gaining prominence in research refers to THS, which consists of the absorption of gases and particles related to tobacco on surfaces such as furniture, walls, and toys, or present in the smoker himself (clothes, hair, and hands). These substances can persist for a period of time ranging from minutes to months.^[9,10]^ Laboratory evidence indicates that exposure to THS negatively affects developing organs and systems^.[11]^ Thus, children and babies with immature respiratory and immune systems are most susceptible to the harmful effects of exposure to THS, which can occur through different routes such as dust ingestion, dermal absorption, and volatile components.^[12]^

Parental information about their children’s exposure to cigarette smoke tends to be inaccurate or underreported. Possible explanations for this situation include denial of exposure by parents, social desirability bias, recall bias, poor awareness of risks, and a lack of knowledge of what constitutes exposure.^[13]^ Accordingly, there is a need to measure parental perceptions of their children’s exposure to tobacco smoke because the most important predictors of health behaviors are perceived vulnerability and perceived severity of a health threat.^[14]^ In an effort to analyze the reasons for children’s continued exposure despite known health risks and the discrepancies between biochemical analyses of children’s exposure and parental reports, the *Parental Perceptions of Children’s Exposure to Tobacco Smoke* (PPE) instrument was developed.^[15]^ Although self-report measures have many shortcomings, there is no objective assessment of individual perception. Thus, the findings of the PPE evaluation may offer valuable perspectives into parental attitudes.

The PPE was initially validated in Hebrew and subsequently translated into English and Portuguese.^[15]^ As the PPE is the only instrument constructed to date to measure adult perceptions about children’s exposure to tobacco smoke, it is important to compare perceptions of exposure and risk as well as behavioral outcomes of smoking. The PPE has already been translated and adapted into Brazilian Portuguese, but a more robust sample measurement properties assessment is needed.^[16]^ Therefore, the aim of this study was to evaluate the measurement properties of the Brazilian version of the PPE.

## 2. Methods

### 2.1. Design and ethical considerations

This is a reliability and validity study of the PPE instrument. The protocol was approved by the Research Ethics Committee of the of the Municipal Department of Health and Civil Defense of Rio de Janeiro, under number CAAE:55824922.6.00005279, and all participants signed an informed consent form. The ethical principles of research involving human beings established by Brazilian Resolution 466/2012 and the Declaration of Helsinki were followed.

### 2.2. Participants

For the development of this study, the convenience recruitment strategy of users linked to the to the primary care of the Coordination of Program Area 5.3, located in the city of Rio de Janeiro, Brazil, was adopted. The recruitment period extended from May to October 2023. The criteria defined for participant eligibility included parents of both genders, smokers or not, with children up to 18 years of age. Individuals who had any type of intellectual disability were excluded. The inclusion of participants in the study was conditioned on their understanding and informed consent regarding the research objectives. After this procedure, interviews were carried out with the recruited participants.

### 2.3. Procedures

Participants completed the Brazilian version of the PPE and a questionnaire to collect sociodemographic data. The PPE, as in the original study,^[15]^ was answered in 2 moments by 10% of randomly selected participants, with an interval of 7 days to evaluate test–retest reliability.

### 2.4. The parental perceptions of children’s exposure to tobacco smoke

**Figure 1.**
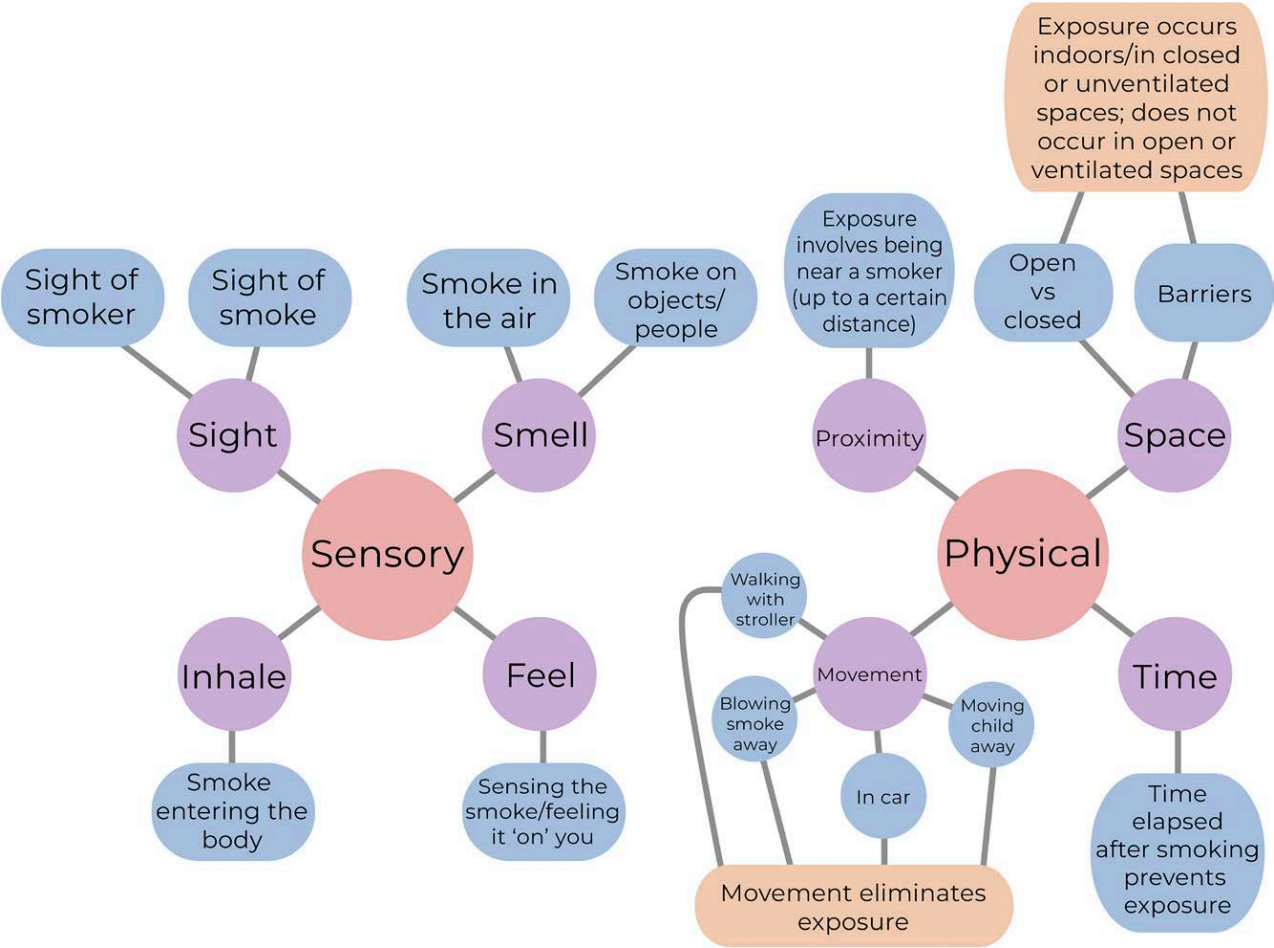
Diagram of the original *parental perceptions of children’s exposure to tobacco smoke* instrument developed from a qualitative study using in-depth interviews.

The PPE, translated and adapted for the Brazilian context,^[16]^ consists of 23 items, 20 of which were scored on a 5-point Likert scale. Seventeen questions assess the degree of exposure, ranging from 1 (“no exposure”) to 5 (“highly exposed”). There are 3 questions that assess confidence in knowledge, ranging from 1 (“not at all”) to 5 (“very confident”). The 3 questions not scored on the Likert scale assess the perception of the time needed for the smoke to dissipate. The original instrument was developed based on a previous qualitative study using in-depth interviews with 65 parents and validated in a subsequent study with 220 Israeli parents. In the analysis of the interviews, a model was developed with 2 general concepts: sensory perception (sight, smell, inhale, and feel) and physical context (proximity, space, movement, and time) (Fig. 1). In these interviews, the interviewees also exposed their uncertainty about their knowledge of the subject.^[9]^

These concepts served as a basis for the development of the original instrument^[15]^ that has the 6 domains, namely secondhand exposure (active exposure through smoking in the home, car or outdoors); third-hand exposure (exposure after someone has moved into the residence or car after several moments); perceived knowledge/certainty (knowledge, certainty and difficulty of response); sensory perceptions (sight and smell); time perceptions (time required for smoke to spread); and 6) distance perceptions (distance traveled by smoke). The PPE sum is obtained by summing all item scores, while the average score is given by dividing the total sum by the number of items answered.

### 2.5. Sample size

A minimum of 100 participants, or 7 times the number of instrument items, is recommended.^[17]^ Considering that the PPE instrument has 23 items, the sample consisted of 161 participants.

### 2.6. Data analysis

Statistical analysis was performed using the R-project software version 4.2.2. Participant characterization data according to sociodemographic variables were analyzed using descriptive statistics. Data quality was checked by ceiling and floor effects and analysis of missing data. The threshold for participants to achieve the highest and lowest scores to define ceiling and floor effects, respectively, was set at 10%. The significance level adopted was 5%.^[18]^

Test–retest stability and internal consistency to assess reliability were verified using the intraclass correlation coefficient (ICC) and the calculation of the Cronbach α coefficient, respectively. In this study, ICC values between 0.10 and 0.39 were considered to represent poor reliability; ICC values between 0.40 and 0.69 moderate reliability; ICC values between 0.70 and 0.99 strong reliability; and ICC values equal to 1 perfect reliability.^[19]^ For internal consistency, Cronbach α values between 0.70 and 0.95 were considered excellent.^[19]^ To evaluate the suitability of the data for factor analysis and ensure the robustness of the results obtained in this study, a correlation matrix was created using the Kaiser–Meyer–Olkin (KMO) test, which considers values >0.50 as acceptable,^[20]^ and the Bartlett sphericity test (*P* < .001), which has the null hypothesis of the absence of correlation between the variables. Thus, the rejection of this hypothesis indicates the existence of some relationships between the variables, justifying the application of factor analysis. To analyze the construct validity of the translated and adapted version, exploratory factor analysis (EFA) was used. Next, the factor extraction method was carried out using principal components, followed by rotation using the Orthogonal Varimax method to assign the weight to each item in each of the retained factors.

Confirmation of the instrument’s structural model was assessed by the commonality of factors through confirmatory factor analysis. To investigate differences in perceptions and attitudes between current smoking, former smoking and nonsmoking parents, an analysis of variance (ANOVA) was conducted. In this analysis, the total score derived from responses to the PPE was analyzed as the dependent variable, while participants’ smoking status was evaluated as an independent variable. If significant results were observed (*P* < .05), Tukey post hoc test was performed.

## 3. Results

### 3.1. Sociodemographic data

Regarding sociodemographic data (Table 1), the majority of participants were female (79.5%), housewives (36.5%), aged between 21 and 40 years old (76.3%), completed undergraduate level (49.1%), had 2 children (38.5%), and never smoked (57.7%). Among parents who declared themselves nonsmokers (n = 92), it was found that 40.2% lived with at least 1 smoker (Table 2). Although the majority of the sample was made up of mothers, this reflects the reality of our country where children remain under maternal care for most of their lives. No ceiling or floor effects were observed, and there was no lost data.

**Table 1:** Sociodemographic characteristics of study participants, which included primary care users with children up to 18 years of age (n = 161).

Variable	*n* (%)
Sex	
Female	128 (79.5)
Male	33 (20.5)
Age range (yr)	
≥20	12 (7.5)
21 to 40	123 (76.3)
41 to 58	26 (16.2)
Smoker	
Yes	50 (30.5)
No	92 (57.7)
Former smoker	19 (11.8)
Number of children	
1	57 (35.4)
2	62 (38.5)
3	29 (18.1)
4	7 (4.3)
>4	6 (3.7)
Number of smokers per household	
0	68 (42.2)
1	70 (43.4)
2	21 (13.2)
>2	2 (1.2)
Education	
None	0 (0)
Primary school	13 (8.1)
High school	54 (33.5)
Undergraduate level	79 (49.1)
Graduate level	15 (9.3)
Occupation	
From home	59 (36.5)
Seller	8 (4.9)
Nursing technician	4 (2.48)
Attendant	4 (2.48)
Others	86 (53.6)

**Table 2:** Distribution of nonsmokers in relation to the home environment.

	Lives with current smoker	Lives with nonsmoker
Nonsmokers (never smoked)	37 (40.2%)	55 (59.8%)
Former smokers (do not currently smoke)	9 (47.3%)	10 (52.7%)

### 3.2. Internal consistency

The analysis of internal consistency using the instrument’s Cronbach α value was 0.92. Detailed analysis of the coefficient revealed that the standardized alpha, which refers to the average of the multiple quadratic correlation coefficients for each item, also presented the same result, with a value equal to 0.92. The average correlation between the items was 0.36, indicating a moderate degree of interrelationship between them, which contributes to the internal consistency of the instrument without suggesting excessive redundancy.

Furthermore, the precision of the Cronbach α coefficient was evaluated using the standard error of estimate found at 0.0081, which indicates a high precision in estimating alpha for this sample. The mean and standard deviation of the items (3.9 ± 0.67, respectively) corroborate the centrality and dispersion of the responses, while the median of the correlation between items (0.36) confirms the observed consistency. The 95% confidence limits for the Cronbach α coefficient calculated using the Feldt and Duhachek methods ranged between 0.90 and 0.94.

### 3.3. Test–retest reliability

To evaluate the test–retest reliability of the applied instrument, 10% of the initial respondents were invited to answer the questionnaire after an interval of 7 days. This subset proved to be representative of the complete sample, with no statistically significant discrepancies in terms of age or smoking habit. Analysis of the correlation between the global scores obtained in the first and second moments revealed an excellent ICC (*R* = 0.91). Furthermore, moderate to strong reliability was observed for the 3 subscales examined (factor 1 = 0.77; factor 2 = 0.60; factor 3 = −0.94; factor 4 = 0.47), although factor 4 demonstrated lower reliability.

### 3.4. Factor analysis

**Figure 2.**
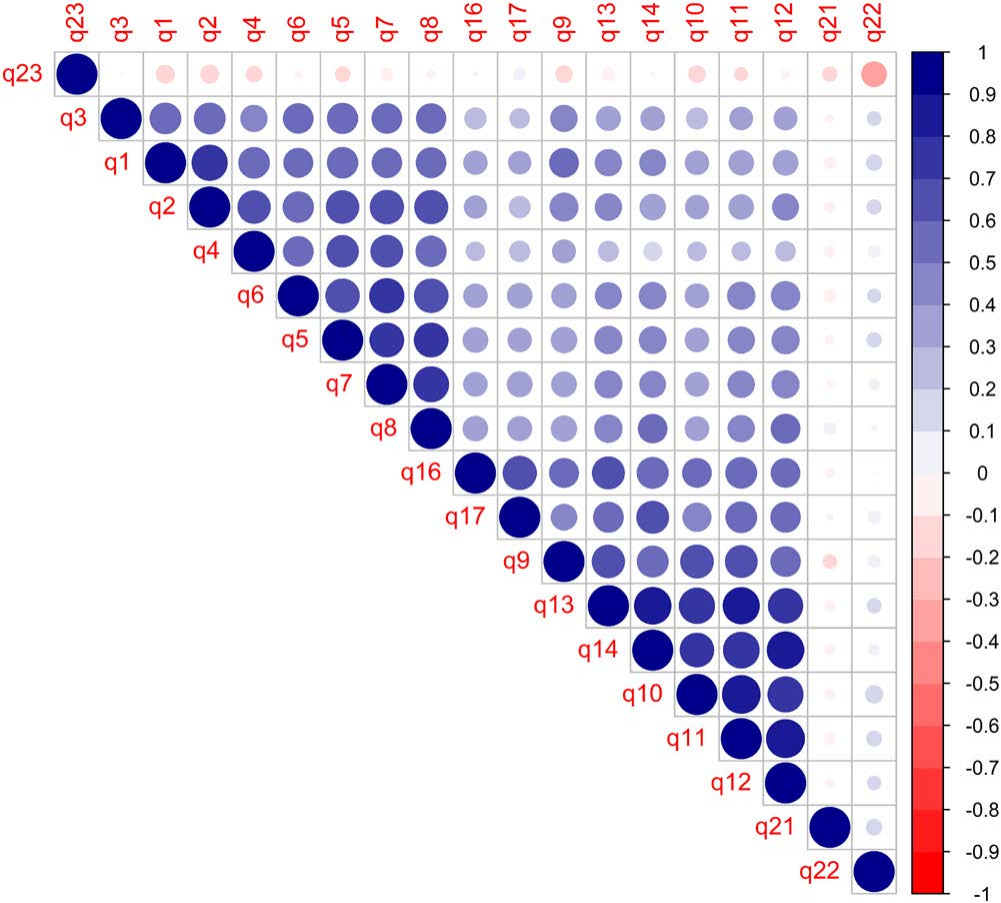
Correlation matrix between the questions (q) on the form. The blue color corresponds to positive correlations between variables, while red color represents negative correlations between them; the intensity of the colors addresses how strong or weak this correlation is.

Analysis of the correlation matrix revealed patterns of moderate to strong correlations between items, suggesting the existence of latent structures that organize the observed variables (Fig. 2).

Items from questions (q) q1 to q7, for example, demonstrate moderate to strong correlations with each other, suggesting that they may be measuring similar aspects of an underlying construct. Although there are variations, this pattern is repeated among other groups of items, such as between q8 and q14 and between q9 and q17, indicating the possibility of multiple factors or dimensions that organize the variables. Specifically, it is observed that items such as q5, q6, and q7 maintain strong correlations not only with each other but also with q8, indicating a possible common dimension. Likewise, the high correlation between items q10 and q14 suggests another potential dimension, possibly distinct from the first. Interestingly, items such as q21, q22, and q23 show low or even negative correlations with most other items, which may indicate that they measure different constructs or are less aligned with the main dimensions identified.

Bartlett sphericity test (*P* < .0001) demonstrated that there is a relationship between the questions.

When applying principal component extraction to the collected data, a varied distribution of the importance of the components was observed. The extraction of eigenvalues >1 resulted in 4 factors. This model explained a total accumulated variance of 69.1% (Table 3).

**Table 3:** Eigenvalues and percentages of accumulated variance.

Eigenvalues	Percentages	Accumulated variance
**2.94**	**43.3%**	**43.3%**
**1.60**	**12.8%**	**56.1%**
**1.23**	**7.51%**	**63.6%**
**1.04**	**5.44%**	**69.1%**
0.96	4.66%	73.7%
0.88	3.86%	77.6%
0.86	3.73%	81.3%
0.82	3.35%	84.7%
0.70	2.44%	87.1%
0.64	2.03%	89.1%
0.59	1.72%	90.9%
0.57	1.61%	92.5%
0.55	1.51%	93.9%
0.50	1.25%	95.2%
0.49	1.20%	96.4%
0.46	1.07%	97.5%
0.43	0.91%	98.4%
0.40	0.78%	99.2%
0.32	0.52%	99.7%
0.25	0.30%	100%

The bold values refer to eigenvalues >1 that explained a total accumulated variance of 69.1%.

After analyzing the communalities, it was found that all variables presented significant values, except for question 15, which obtained a communality of 0.29. This lower-than-expected value suggests that question 15 does not contribute adequately to the variance explained by the model. Therefore, it was decided to exclude question 15 from the study and the analysis was redone.

The results obtained reveal significant communalities for all variables analyzed, ranging from 0.58 to 0.87, which indicates an excellent representation of the variables by the extracted factors.

In continuation of the factor analysis, the Orthogonal Varimax method was used to improve the interpretation of the extracted factors (Table 4).

**Table 4:** Rotated factor loadings.

Questions	Factor 1	Factor 2	Factor 3	Factor 4
q1	0.243	**0.726**	0.224	0.194
q2	0.211	**0.824**	0.175	0.130
q3	0.190	**0.698**		
q4		**0.792**		
q5	0.278	**0.800**		
q6	0.303	**0.759**		
q7	0.267	**0.820**		
q8	0.315	**0.796**		−0.197
q9	**0.645**	0.325	0.208	0.287
q10	**0.854**	0.131	0.233	0.112
q11	**0.908**	0.198	0.111	
q12	**0.838**	0.276		
q13	**0.854**	0.261		
q14	**0.865**	0.250		
q16	**0.715**	0.214	−0.135	
q17	**0.700**	0.214	−0.162	−0.220
q21			0.193	**−0.888**
q22			**0.733**	−0.201
q23			**−0.837**	

The bold values refer to the values selected for each question using rotated factor loadings.

For the EFA carried out in this study, it is worth highlighting that q18, q19, and q20 were excluded due to the predominance of “I don’t know” answers among the participants. Specifically, it was observed that 81.9% of participants selected the “I don’t know” option for q18, while 73.2% opted for the same answer for q19 and q20. This disproportionate distribution of responses made it impossible to include these questions in the factor analysis because the presence of these inconclusive responses could distort the factor structure underlying the measurement instrument.^[21]^ Therefore, in accordance with established statistical and methodological criteria, these questions were excluded from the factor analysis, thus guaranteeing the integrity and interpretability of the results obtained.

**Figure 3.**
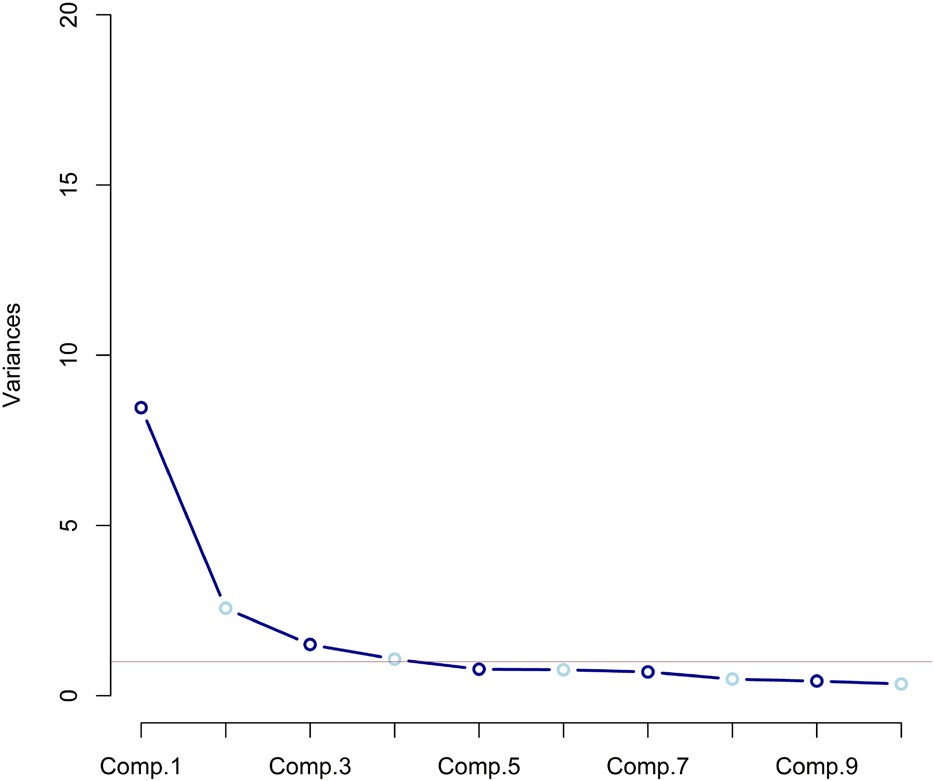
Scree plot where the variances explained by each component are shown on the Y axis, while the main components numbered sequentially are shown on the X axis.

Performing factor analysis with 19 items (three were excluded because they were not on a Likert scale, and question 15 was excluded because it had low communality) resulted in 4 factors with eigenvalues >1. Supporting the choice of retaining 4 factors, the scree plot analysis was performed (Fig. 3). On the vertical axis (Y axis), we have the variance explained by each component, while on the horizontal axis (X axis) the principal components are represented sequentially numbered. From the fourth principal component onwards, a decrease in the explained variance is observed, which then stabilizes. This stable trend suggests that most of the data variability can be captured by the first 4 principal components, with the additional components contributing marginally to the total variance. This model explained a total accumulated variance of 71.6%. The factors are presented in Table 5.

**Table 5:** Factor labels and Cronbach scores.

Factor	Label	Description	Items	Cronbach α	Eigenvalue	Accumulated variance
1	Third-hand exposure and sensory exposure	Exposure after someone has smoked in the car or at home at various times. Sight and smell.	q9 to q17	0.93	2.90	44.52%
2	secondhand exposure	Exposure from active smoking in the car, at home, and outdoors	q1 to q8	0.92	1.60	58.03%
3	Certainty	How the respondent felt when answering the questionnaire	q22 to q23	0.61	1.22	65.93%
4	Perceived knowledge	How the respondent sees themselves in relation to the subject of the study	q21	–	1.04	71.58%

To validate and confirm the factorial structure identified in the exploratory stage, a combined factor analysis was carried out. The model was adjusted using robust maximum marginal likelihood estimation and included 7 factors as identified in the EFA. The results indicated a good fit of the model to the data, with a significant chi-square test (χ² = 358,657, df = 147, *P* < .001), indicating that the proposed model adequately explained the observed correlation matrix. The standardized factor loadings were significant (*P* < .05) for most items, indicating that they were well represented by the latent factors. Furthermore, covariances between factors were consistent with theoretical expectations, suggesting the presence of significant associations between factors. The variances of the observed variables and latent factors were adequate, with standardized factor variances set at 1.

### 3.5. Current smoker versus nonsmoker

The distribution of average scores obtained on the PPE instrument among the groups of participants categorized as current smokers, former smokers, and nonsmokers is summarized in Table 6. These average scores are the result of the sum of the answers to the PPE questions divided by the total number of questions scored on the Likert scale, that is, 19 for each of the 7 factors analyzed. Current smokers (n = 50) had a total PPE sum of 138.8 and a mean of 2.77, with specific distributions per factor varying between 7.2 (factor 4) and 71.7 (factor 2), indicating different perceptions regarding the different aspects assessed by the questionnaire. Former smokers (n = 19) demonstrated a total PPE sum of 71 and a mean of 3.73, with factor scores varying between 3.05 (factor 4) and 36.9 (factor 2), indicating a variation in perceptions among individuals who have stopped smoking. On the other hand, nonsmokers (n* *= 92), with a total PPE sum of 361.5 and a mean of 3.92 on the PPE, exhibited average scores close to former smokers in all factors, ranging from 11.9 (factor 4) to 171.5 (factor 2).

**Table 6:** Parental perceptions of children’s exposure to tobacco smoke (PPE) scores of current smokers, former smokers, and nonsmokers.

Classification	Current smokers (n = 50)		Former smokers (n = 19)		Nonsmokers (n = 92)	
	Total sum	Mean*	Total sum	Mean*	Total sum	Mean*
PPE value	138.8	2.77	71	3.73	361.5	3.92
Factor 1	47.9	0.95	28.2	1.48	157.7	1.71
Factor 2	71.7	1.43	36.9	1.94	171.5	1.86
Factor 3	17.2	0.34	8.15	0.42	31.05	0.66
Factor 4	7.2	0.14	3.05	0.16	11.9	0.13

* These average scores are the result of the sum of the answers to the PPE questions divided by the total number of questions scored on the Likert scale, that is, 19 for each of the 7 factors analyzed.

The ANOVA showed statistical significance, which indicates that at least 1 of the groups differs from the others in terms of mean score. Specifically, Tukey post hoc test identified significant differences between the groups of current smokers and former smokers, where smokers had significantly lower mean scores compared to former smokers, with a mean difference of −18.6 (95% CI: −23.9 to −14.4, *P* < .0001). Furthermore, a significant difference was observed between nonsmokers and smokers, with nonsmokers showing significantly higher mean scores, indicating a mean difference of 22.6 (95% CI: 19.3 to 26.7, *P* < .0001). However, the comparison between nonsmokers and former smokers revealed no statistically significant differences, with a mean difference of 3.05 (95% CI: −1.89 to 7.97, *P* = .31) (see the Portuguese version of the PPE instrument translated and adapted to the Brazilian context as Supplementary File, Supplemental Digital Content, http://links.lww.com/MD/N752).

## 4. Discussion

This study investigated the measurement properties of the Brazilian version of the PPE, an instrument created in Israel to assess parents’ perceptions of their children’s exposure to tobacco smoke. Given that much of the exposure is caused by parents themselves, it is extremely important to understand their perception in order to use effective strategies to reduce exposure and harm caused by tobacco smoke. As in the original version, the results of this study showed adequate reliability and validity.^[15]^ The absence of ceiling and floor effects indicated good acceptability of the instrument.

Reliability and validity analyzes have been widely recommended to investigate the ability to reproduce a result cohesively in time and space and verify whether the instrument really measures what it purports to measure.^[22]^ The internal consistency of the instrument (Cronbach α = 0.92) demonstrated values above 0.70, therefore being considered adequate.^[19]^ Furthermore, the instrument proved to be stable when comparing applications with an interval of 1 week.^[19]^ The test–retest stability coefficient showed remarkable stability (ICC = 0.91) in responses over time. The lower reliability of factor 4 (ICC = 0.47) can be attributed to the nature of the question that makes up factor 4, as it explores the participant’s perception of their knowledge regarding the topic studied, suggesting that initial exposure to the questionnaire may have influenced their subsequent self-assessment. After a factorial reanalysis excluding the item from factor 4, a marginal decrease in the total variance explained was noted. Therefore, it was decided to maintain the original structure of factor 4.

When comparing the factorial structure found in the study with the original, we found similarities in the dimensions of both versions but with different groupings. It is worth mentioning that the factorial analysis of our study was performed with 19 questions. After analyzing the commonalities, it was necessary to remove question 15 because it was not connected with the others. Although there is no consensus on the number of items to be extracted, the decision to retain 4 factors was based on the main criteria recommended by the literature:^[23]^ eigenvalue criterion > 1, aided by the scree plot. We additionally used the accumulated variance criterion where the level of 60% is considered acceptable.^[21]^ One point to consider is that the Brazilian version has a structure with 22 items and 4 domains and a total accumulated variance of 71.6%, which is different from the original version as the latter has 6 domains and a total accumulated variance of 76.3%. Despite this, the confirmatory factor analysis provided robust evidence of the factor structure underlying the measurement instrument, thus validating the factor structure identified in the EFA. Of note, we chose to keep all questions those not scored in Likert, as future studies can improve knowledge about these questions and enable new investigations by carrying out a factor analysis.

The results of the analysis of variance suggest that the perceptions assessed by the PPE instrument differ significantly between current smokers, former smokers, and those who have never smoked. As in the original study, current smokers have the lowest average scores and nonsmokers the highest.^[15]^ The absence of a significant difference between nonsmokers and former smokers may indicate similarities in the perceptions or behaviors of these 2 groups in relation to the phenomenon studied. The analysis suggests the importance of considering current and past smoking status in assessments and interventions related to parents’ perceptions of their children’s exposure to tobacco smoke.

In this way, the statistical analysis carried out highlights significant differences in the average scores between current smoking, former smoking, and nonsmoking parents. There is important information from a social point of view, especially in the context of the impact of smoking and health-related behaviors on parental perceptions and attitudes. These results suggest that smoking behavior can negatively impact parents’ perceptions of issues relevant to the study, possibly due to their own involvement in smoking. This can potentially limit perceptions about the risks associated with exposure to tobacco smoke and the importance of adopting healthy behaviors.^[13]^

Parents who smoke had the lowest mean scores, which may indicate less awareness or concern about the issues assessed by the PPE instrument. This can have direct implications for health promotion in the family environment, influencing not only the health of parents but also potentially affecting the health of children.^[24]^ On the other hand, nonsmoking parents, by presenting higher average scores, possibly demonstrate greater awareness or engagement with the issues assessed, which may reflect a more significant appreciation of health and well-being.^[4]^ Interestingly, the lack of a significant difference between the mean scores of former smokers and nonsmokers may indicate that individuals who have managed to quit smoking are possibly undergoing a reevaluation of their health-related attitudes and behaviors towards smoking, aligning more closely with the perceptions and behaviors of individuals who have never smoked. This suggests that smoking cessation may not only bring direct benefits to the physical health of former smokers but also contribute to a positive change in terms of attitudes and behaviors related to health and well-being in the family context.^[8]^

### 4.1. Limitations

Some limitations of our study should be noted. First, Brazil is a country of continental dimensions, containing 5 geographic regions with profound socioeconomic and cultural differences between them; therefore, there is a need to carry out further investigations to obtain more evidence of the validity and reliability of the PPE instrument, using representative samples from different regions of Brazil with a larger number of individuals for retesting. The Brazilian version had 1 item excluded and its application and scoring method changed. As the original scale was not subjected to the processes of translation, adaptation, and validation in other cultures, comparison of our results is not yet possible.

## 5. Conclusions

Our findings provide evidence that the PPE version translated and adapted for the Brazilian context is a reliable and valid instrument. It allows measuring parents’ perceptions about children’s exposure to tobacco smoke in 4 specific factors: third-hand exposure and sensory exposure, secondhand exposure, certainty, and perceived knowledge. Taken together, this can help us understand how parents cope with exposure to tobacco smoke. Finally, the study results highlight the need for educational interventions that consider the differences in perceptions between current smokers, former smokers, and nonsmokers in an attempt to modify parental behavior and reduce children’s exposure to tobacco smoke.

## Author contributions

**Conceptualization:** Maria Alice Santos Tavares, Agnaldo José Lopes.

**Data curation:** Maria Alice Santos Tavares, Vicki Myers, Leandro Alberto Calazans Nogueira.

**Formal analysis:** Maria Alice Santos Tavares, Agnaldo José Lopes.

**Funding acquisition:** Agnaldo José Lopes.

**Investigation:** Vicki Myers, Leandro Alberto Calazans Nogueira.

**Methodology:** Maria Alice Santos Tavares, Vicki Myers, Leandro Alberto Calazans Nogueira, Agnaldo José Lopes.

**Project administration:** Maria Alice Santos Tavares.

**Supervision:** Agnaldo José Lopes.

**Validation:** Maria Alice Santos Tavares, Agnaldo José Lopes.

**Writing – original draft:** Maria Alice Santos Tavares, Vicki Myers, Leandro Alberto Calazans Nogueira, Agnaldo José Lopes.

**Writing – review & editing:** Maria Alice Santos Tavares, Vicki Myers, Leandro Alberto Calazans Nogueira, Agnaldo José Lopes.

## Supplementary Material


