# Supplementary material for: Evaluation of the measurement properties of the parental perceptions of children’s exposure to tobacco smoke instrument translated and adapted to the Brazilian context
Source: Medicine (Baltimore). 2024 Oct 25;103(43):e40147. doi: 10.1097/MD.0000000000040147 (PMC11521034; doi:10.1097/MD.0000000000040147)
Supplement: Supplementary file 1 [file medi-103-e40147-s001.pdf]

Percepção dos pais sobre o questionário de exposição (PPE)

A. Neste questionário você será apresentado a várias situações mostrando fumantes e crianças.

Em qual grau você acha que a criança na imagem está exposta à fumaça do cigarro?

(Em qual grau a fumaça atinge a criança)

Classifique sua resposta entre 1 =nenhuma exposição e 5= altamente exposta.

1. 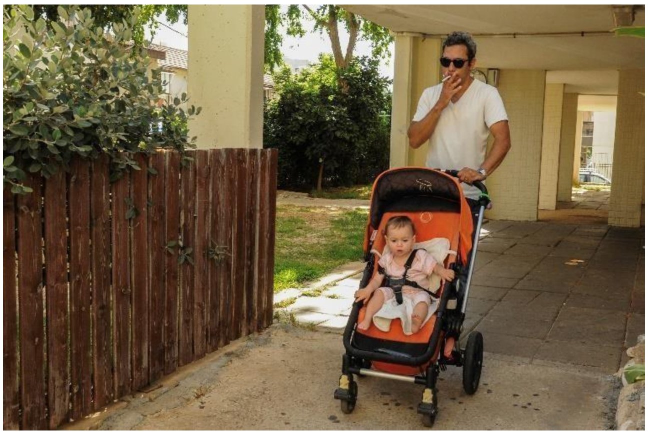

Não exposta (1) (2) (3) (4) (5) Altamente exposta

2. 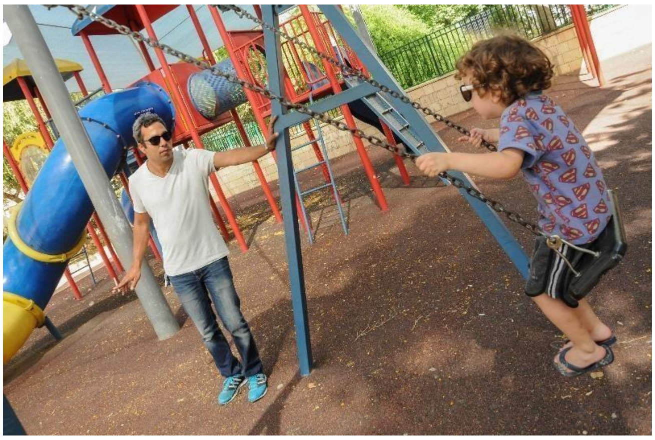

Não exposta (1) (2) (3) (4) (5) Altamente exposta

Scanned with CamScanner

3 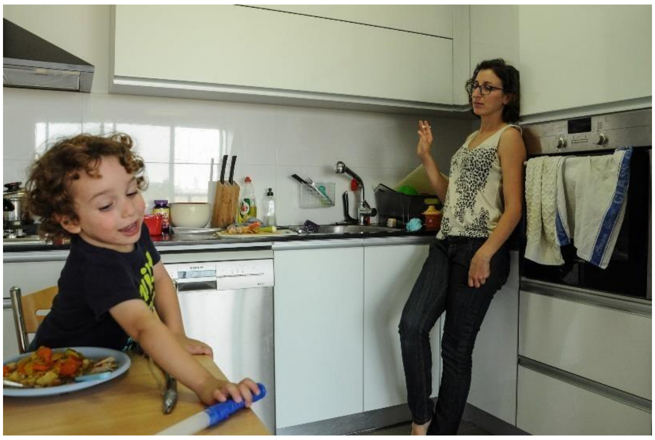

Não exposta (1) (2) (3) (4) (5) Altamente exposta

4. 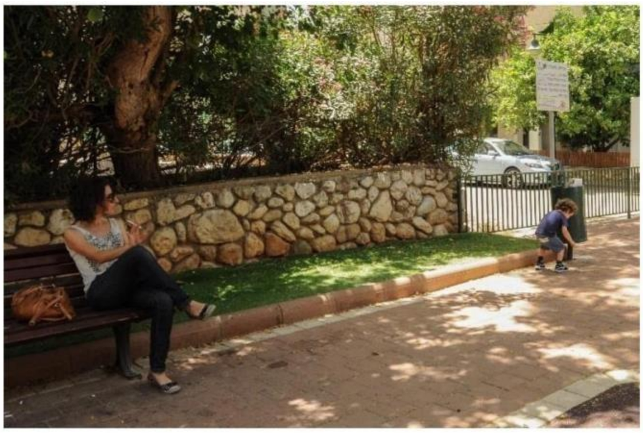

Não exposta (1) (2) (3) (4) (5) Altamente exposta

5 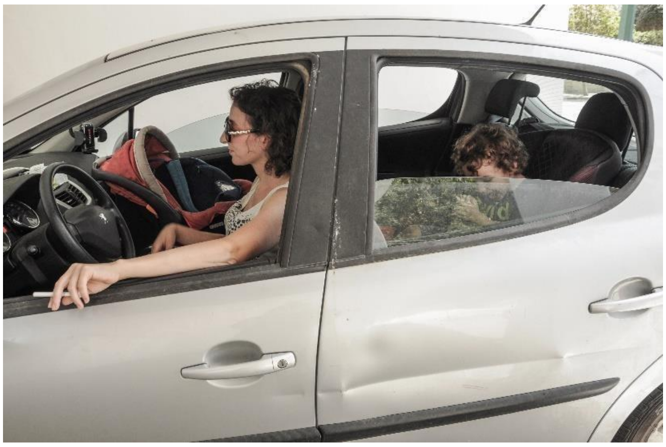

Não exposta (1) (2) (3) (4) (5) Altamente exposta

Scanned with CamScanner

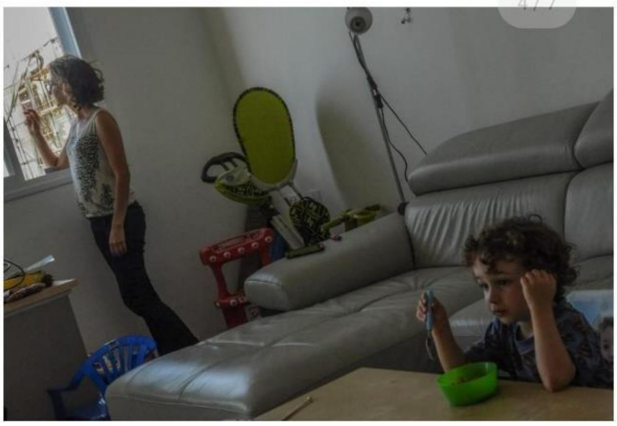

6.

Não exposta (1) (2) (3) (4) (5) Altamente exposta

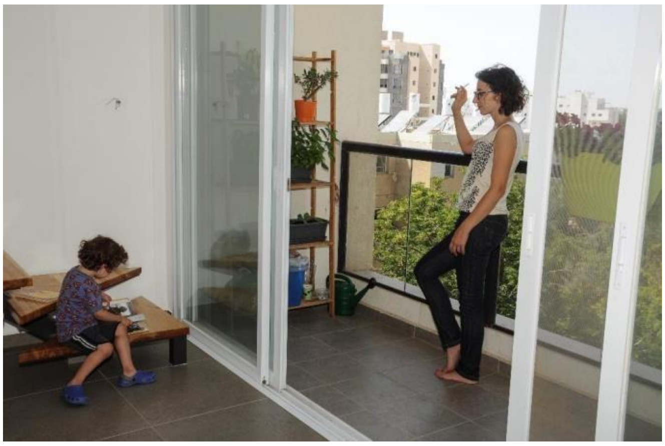

7

Não exposta (1) (2) (3) (4) (5) Altamente exposta

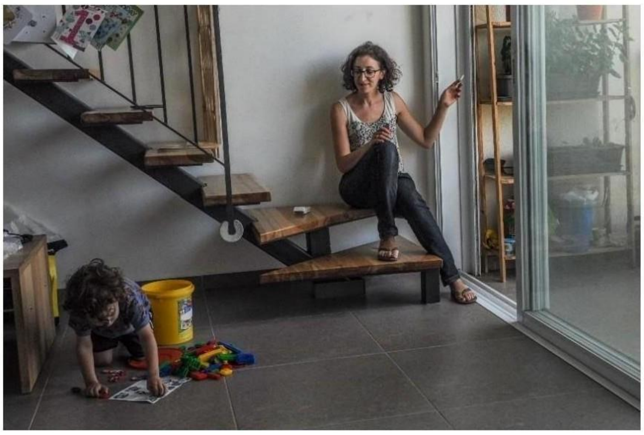

8.

Não exposta (1) (2) (3) (4) (5) Altamente exposta

Scanned with CamScanner

B. Nas perguntas seguintes as situações serão descritas sem imagens.

Por favor, avalie, em sua opinião, o grau de exposição da criança descrita a fumaça do cigarro. (Em qual grau a fumaça atinge a criança)

Classifique sua resposta entre 1 =nenhuma exposição e 5= altamente exposta.

9. A criança está na cozinha, alguém está fumando na área de serviço ao lado

Não exposta (1) (2) (3) (4) (5) Altamente exposta

10. A criança está em um quarto onde alguém fumou 12 horas atrás

Não exposta (1) (2) (3) (4) (5) Altamente exposta

11. A criança está em um quarto onde alguém fumou 2 horas atrás

Não exposta (1) (2) (3) (4) (5) Altamente exposta

12. A criança está em um quarto onde alguém fumou 30 minutos atrás

Não exposta (1) (2) (3) (4) (5) Altamente exposta

13. A criança está em um carro onde alguém fumou 1 hora atrás

Não exposta (1) (2) (3) (4) (5) Altamente exposta

14. A criança está em um carro onde alguém fumou 20 minutos atrás

Não exposta (1) (2) (3) (4) (5) Altamente exposta

15. A criança está no parquinho e sente o cheiro da fumaça do cigarro mas não vê o fumante

Não exposta (1) (2) (3) (4) (5) Altamente exposta

16. A criança está no parquinho e vê a sua mãe fumando e consegue sentir o cheiro da fumaça

Não exposta (1) (2) (3) (4) (5) Altamente exposta

Scanned with CamScanner

17. Qual a proporção da fumaça do tabaco é invisível?

( ) 20% ( )50% ( )80% ( ) NÃO SEI

18. Depois de fumar em casa, quanto tempo leva para a casa ficar livre de fumaça?

( ) 0-1 hs, ( ) 2-3 hs, ( ) 4-12 hs, ( )13-24 hs, ( ) 25-48 hs,
( ) 48 hs a 1 semana, ( ) 1 mês - vários meses, ( ) anos ou nunca

( ) não sei

19. Depois de fumar no carro, quanto tempo leva para o carro ficar livre de fumaça?

( ) 0-1 hs, ( ) 2-3 hs, ( ) 4-12 hs, ( )13-24 hs, ( ) 25-48 hs,
( ) 48 hs a 1 semana, ( ) 1 mês - vários meses, ( ) anos ou nunca

( ) não sei

20. Você considera que sabe o suficiente sobre o assunto fumante passivo?

De jeito nenhum (1) (2) (3) (4) (5) muito

21. Você se sentiu com as suas respostas?

De jeito nenhum (1) (2) (3) (4) (5) muito

22. Você achou difícil responder ao questionário?

De jeito nenhum (1) (2) (3) (4) (5) muito

Scanned with CamScanner
